# Supplementary material for: Enhancing photovoltages at p-type semiconductors through a redox-active metal-organic framework surface coating
Source: Nat Commun. 2020 Nov 16;11:5819. doi: 10.1038/s41467-020-19483-5 (PMC7669860; doi:10.1038/s41467-020-19483-5)
Supplement: Supplementary file 1 — Supplementary Information [file 41467_2020_19483_MOESM1_ESM.pdf]

Supplementary Information for

Enhancing Photovoltages at p-Type Semiconductors Through a Redox-active Metal-Organic Framework Surface Coating

Anna M. Beiler, Brian D. McCarthy, Ben A. Johnson, and Sascha Ott*

Department of Chemistry, Ångström Laboratory, Uppsala University, Box 523, 75120 Uppsala, Sweden

Supplementary Figures

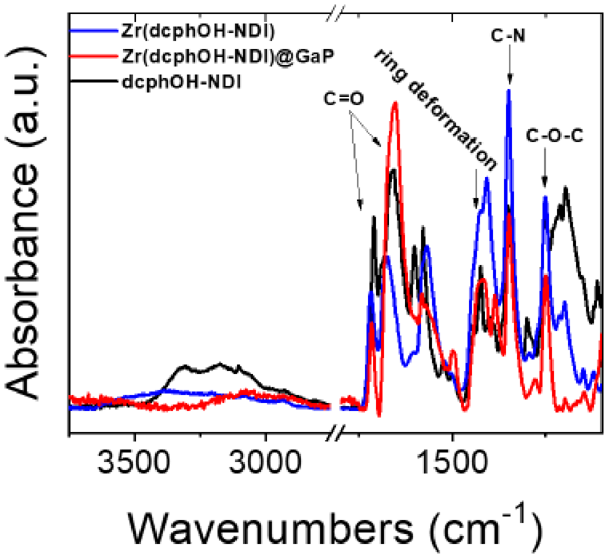

Supplementary Figure 1. ATR-FTIR absorbance spectrum of bulk NDI powder (black), Zr(NDI) powder (blue) and of Zr(NDI)|TiO_2@GaP (red).

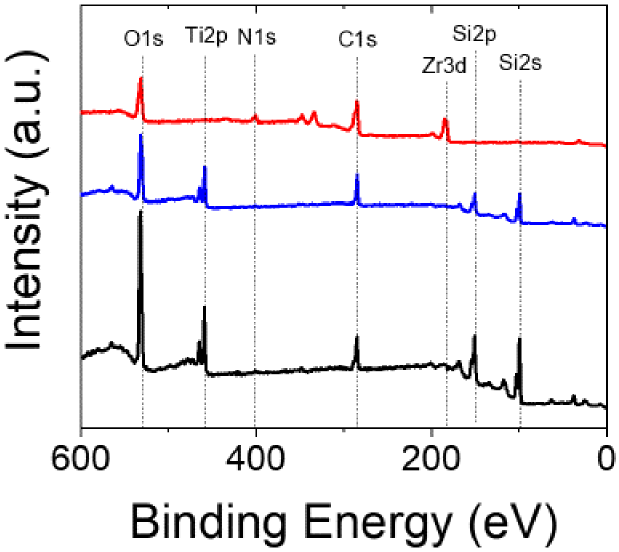

Supplementary Figure 2. Survey XPS spectra of TiO_2@Si (black), NDI | TiO_2@Si (blue), and Zr(NDI) | TiO_2@Si (red).

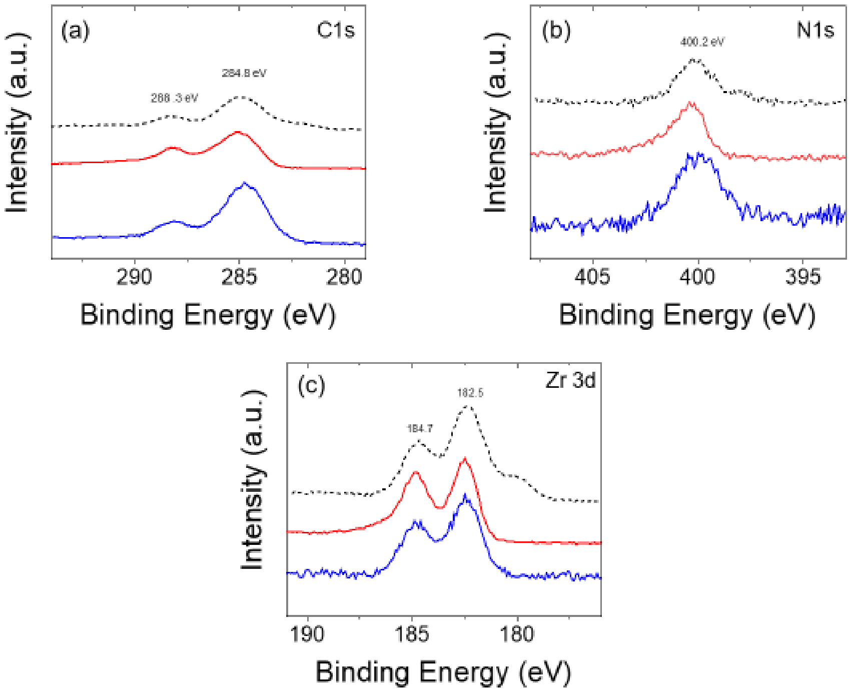

Supplementary Figure 3. High-resolution XPS spectra of the (a) C1s, (b) N1s, and (c) Zr3d regions of Zr(NDI) | TiO_2@Si (blue), Zr(NDI) | TiO_2@GaP (red), and the bulk MOF Zr(NDI) (black dashed).

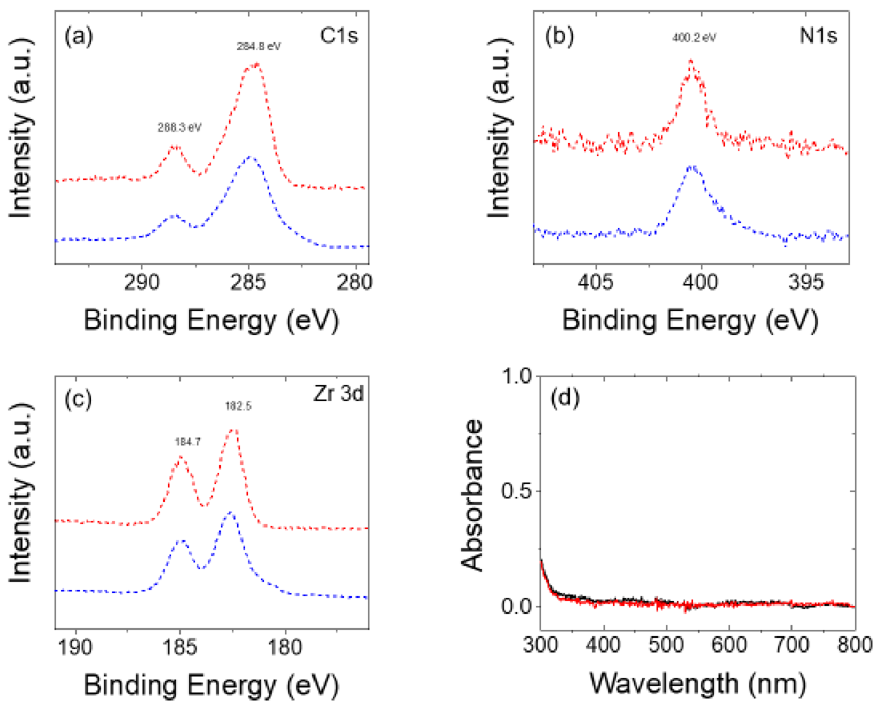

Supplementary Figure 4. (a-c) High-resolution XPS spectra of the (a) C1s, (b) N1s, and (c) Zr3d regions of Zr(NDI)|TiO_2@Si (blue) and Zr(NDI)|TiO_2@GaP (red) after photoelectrochemical operation, as well as (d) UV-Vis spectra of the electrolyte before (black) and after (red) photoelectrochemical operation of Zr(NDI)|TiO_2@Si.

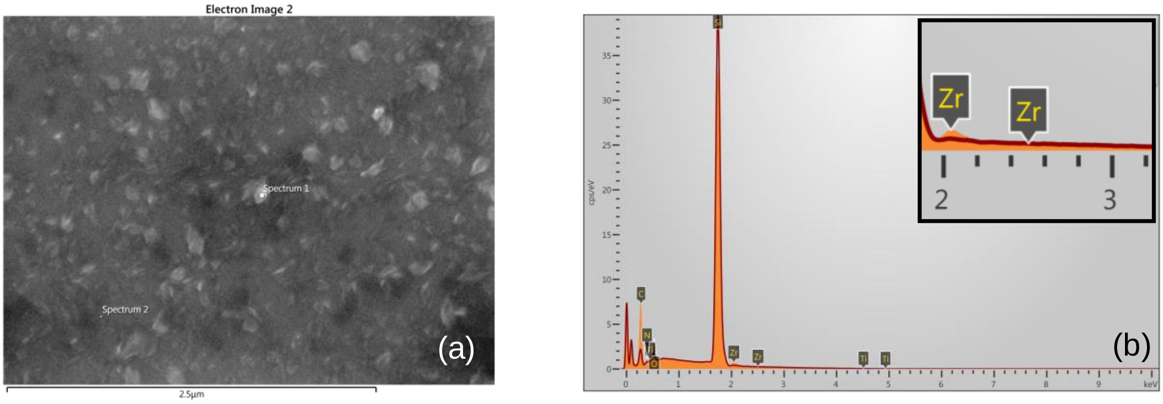

Supplementary Figure 5. Representative SEM-EDX results for Zr(NDI)│TiO_2@SC. (a) SEM image of Zr(NDI)│TiO_2@Si sample. (b) EDX data of Spectrum 1 (orange data) and Spectrum 2 (red line) from (a). Detailed maps could not be collected due to charging of the semiconductor│MOF constructs (already evident here in the blurring of the image on short time scales). However, line scan, point & id, and map data collected at short time scales show Zr and N present across the sample in a 1:2 ratio. While the absolute counts were higher in areas where a clear crystal was seen (such as Spectrum 1), the consistent ratio across the sample suggests a thin MOF film with good surface coverage along with some larger crystals that are more visible by SEM.

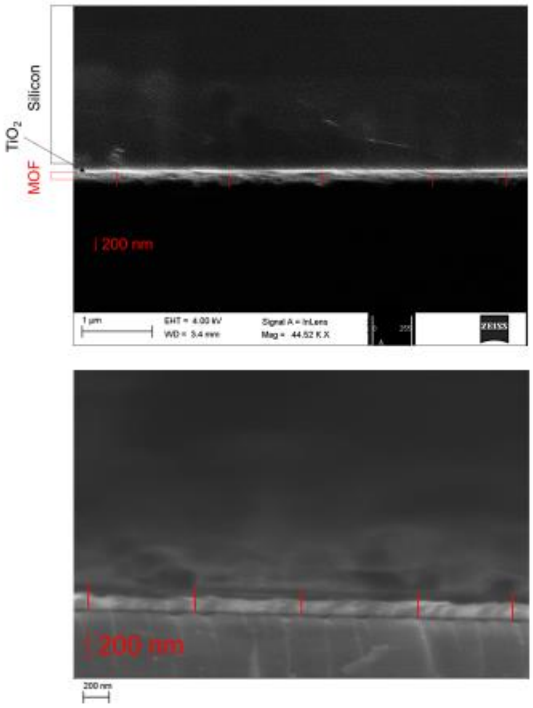

Supplementary Figure 6. SEM cross-sectional images of Zr(NDI)│TiO_2@Si, showing a thickness of 100-200 nm.

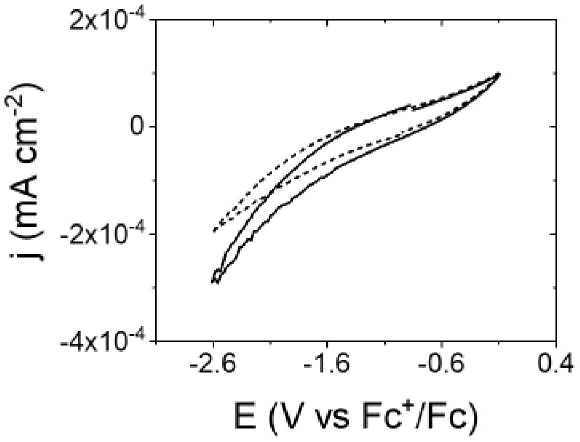

Supplementary Figure 7. Cyclic voltammograms of TiO_2@Si working electrodes in the dark (dashed) and under AM 1.5 illumination (solid) at a scan rate of 100 mV s^{-1} with 0.5 M $LiClO_4$ in DMF as the supporting electrolyte.

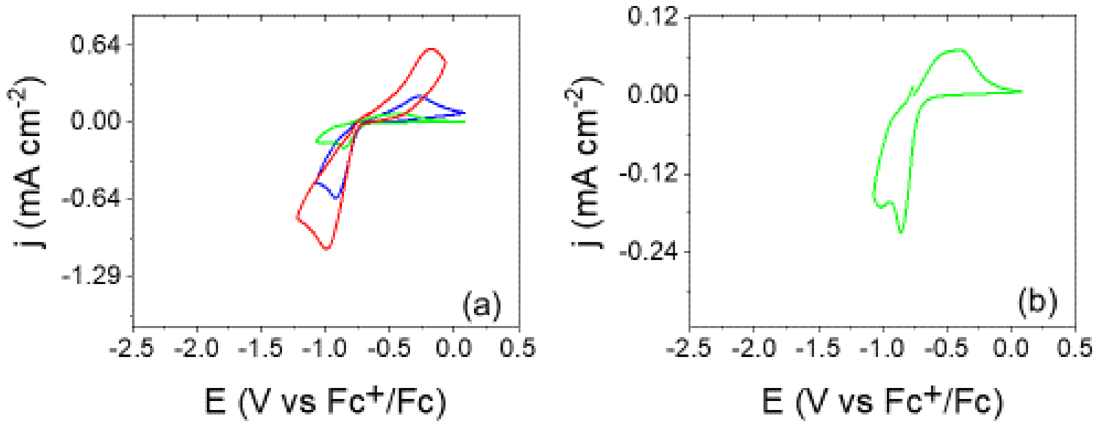

Supplementary Figure 8. Cyclic voltammograms of Zr(NDI)|TiO_2@Si working electrodes under AM 1.5 illumination at (a) scan rates of 100 (red), 25 (blue), and 5 (green) mV s^{-1} and (b) 5 mV s^{-1} only with 0.5 M $LiClO_4$ in DMF as the supporting electrolyte.

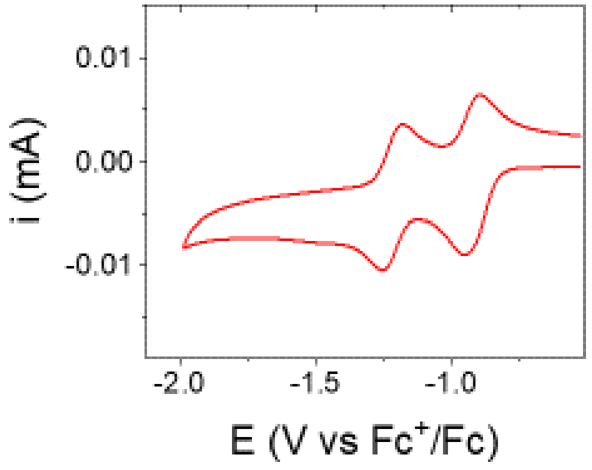

Supplementary Figure 9. Cyclic voltammogram of dcphOH-NDI (the NDI linker used in this work) dissolved in solution at a scan rate of 100 mV s^{-1} with 0.5 M $LiClO_4$ in DMF as the supporting electrolyte.

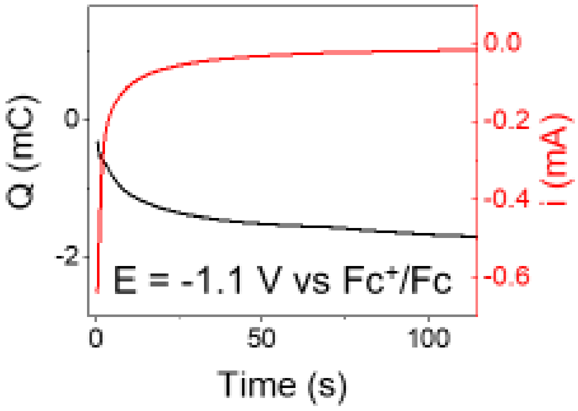

Supplementary Figure 10. Controlled potential electrolysis (red) and total charge passed (black) for a Zr(NDI)|TiO_2@Si working electrode at a potential of -1.1 V vs $Fc^{+/0}$ with 0.5 M $LiClO_4$ in DMF as the supporting electrolyte under AM1.5 illumination.

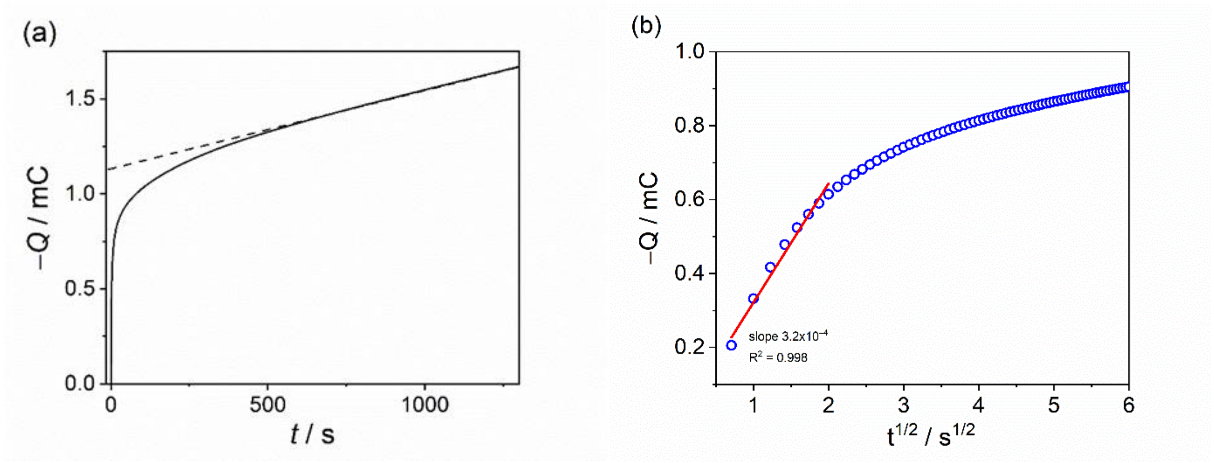

Supplementary Figure 11. (a) Potential step chronocoulometry of Zr(NDI)@FTO film in DMF using 0.5 M $LiClO_4$ as the supporting electrolyte. The potential was stepped to −1.1 V vs. $Fc^{+/0}$. The total charge passed after complete reduction of the film was measured after subtraction of residual background current (dotted line); Γ^0 = 1.2 x 10^{-8} mol cm^{-2}. (b) Plot of charge *vs.* $\sqrt{t}$. The slope was used to compute D_{app} = 6.4 x 10^{-10} $cm^2\,s^{-1}$.

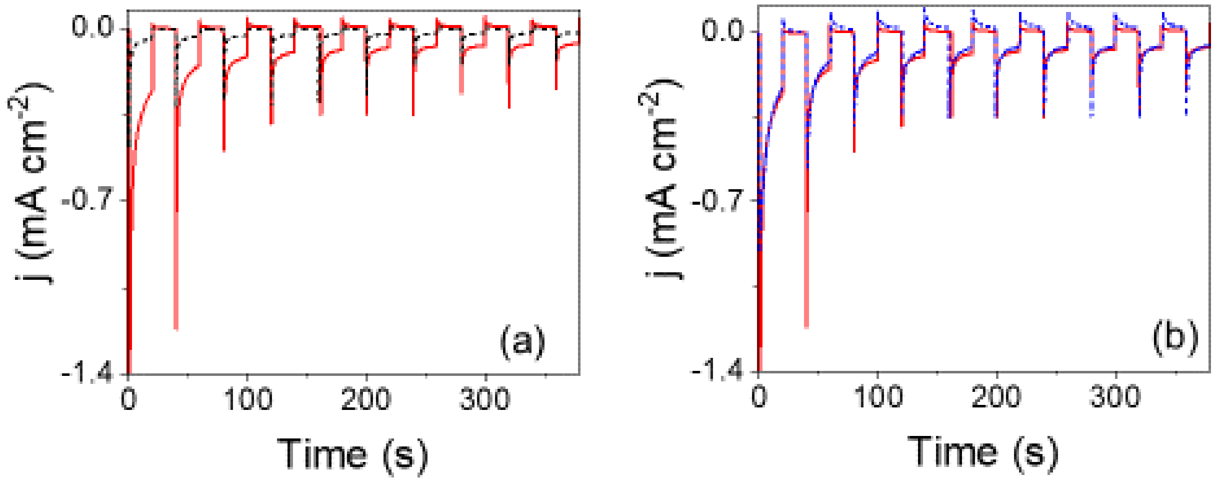

Supplementary Figure 12. Chonoamperomograms of Zr(NDI)│TiO_2@Si (a) in the presence of (red) and absence (black dashed) of 1 mM tris(2,2'-bipyridine)cobalt(III) tris(hexafluorophosphate) and (b) in the presence of unstirred (red) and stirred (blue dashed) 1 mM tris(2,2'-bipyridine)cobalt(III) tris(hexafluorophosphate) under chopped light illumination at a potential of -1.1 V vs $Fc^{+/0}$ with 0.5 M $LiClO_4$ in DMF as the supporting electrolyte.

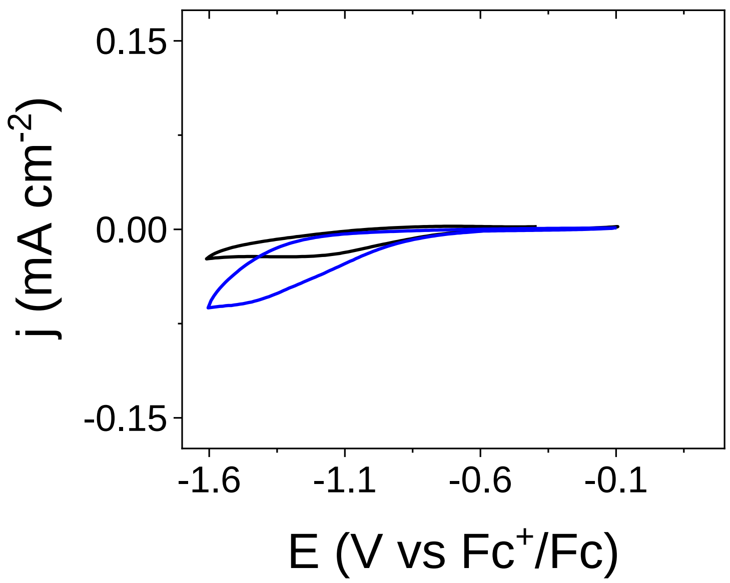

Supplementary Figure 13. Cyclic voltammograms of TiO_2@GaP (black) and NDI│TiO_2@GaP (blue) working electrodes under AM 1.5 illumination at a scan rate of 100 mV s^{-1} with 0.5 M $LiClO_4$ in DMF as the supporting electrolyte.

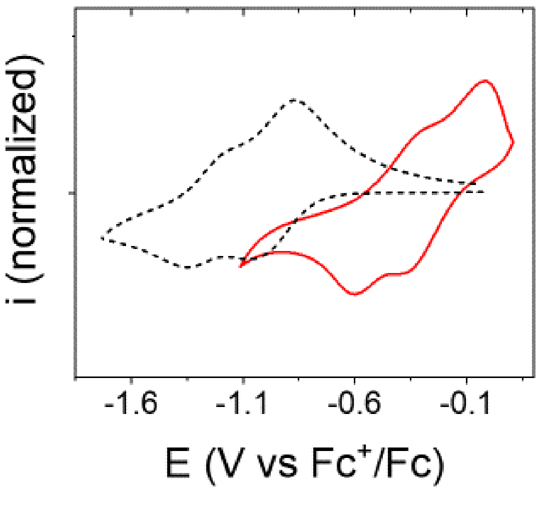

Supplementary Figure 14. Cyclic voltammograms of Zr(NDI)@FTO (black) with 0.8 M KPF_6 in DMF as the supporting electrolyte (data previously published in reference 1) and Zr(NDI)│TiO_2@GaP (red) working electrodes under AM 1.5 illumination with 0.5 M $LiClO_4$ in DMF as the supporting electrolyte. All scans were collected at a scan rate of 100 mV s^{-1}. Data are normalized for comparison.

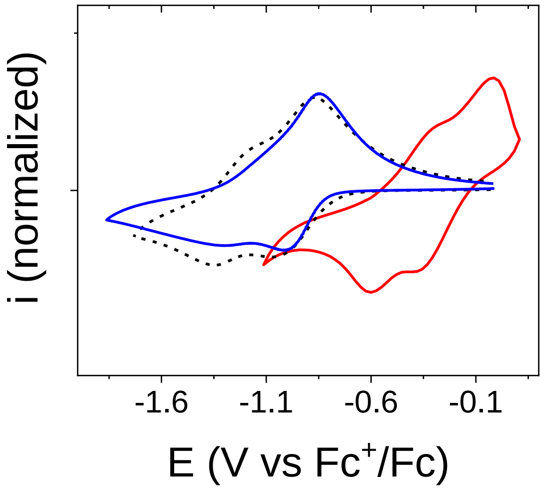

Supplementary Figure 15. Cyclic voltammograms of Zr(NDI)@FTO (black dashed) with **0.8 M KPF_6** in DMF as the supporting electrolyte (data previously published in reference 1) and Zr(dcphOH-NDI@FTO) (blue) with **0.5 M $LiClO_4$** in DMF as the supporting electrolyte, as well as Zr(dcphOH-NDI)|TiO_2@GaP) (red) working electrodes under AM 1.5 illumination with **0.5 M $LiClO_4$** in DMF as the supporting electrolyte. All scans were collected at a scan rate of 100 mV s^{-1}. Data are normalized for comparison.

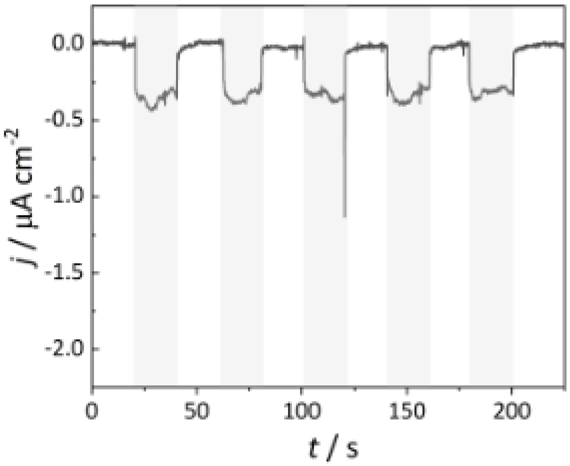

Supplementary Figure 16. Chonoamperomogram of Zr(NDI)@FTO at a bias potential of -1.2 V vs $Fc^{+/0}$ with 0.5 M $LiClO_4$ in DMF as the supporting electrolyte under chopped light illumination (100 mW cm^{-2}, shaded areas). The difference in current between dark and light conditions is <0.5 µA cm^{-2}, illustrating that the photocurrent response for the MOF immobilized at Si or GaP (Figure S12) is dominated by the underpinning semiconductor.

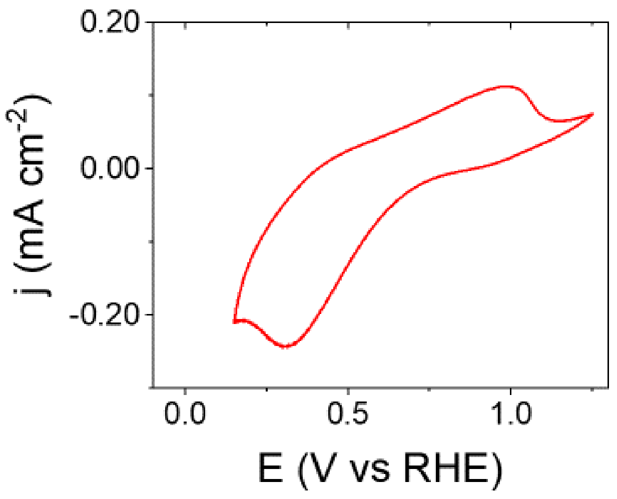

Supplementary Figure 17. Cyclic voltammogram of Zr(dcphOH-NDI)|TiO_2@GaP working electrode with 0.5 M $LiClO_4$ in water as the supporting electrolyte (pH = 8.5) at a scan rate of 100 mV s^{-1} under AM 1.5 illumination.

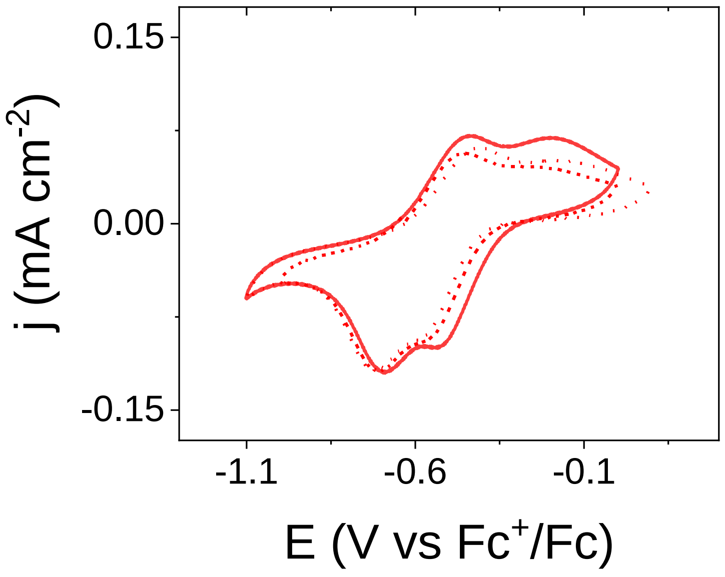

Supplementary Figure 18. Cyclic voltammograms of Zr(dcphOH-NDI)|TiO_2@GaP working electrodes at 1-sun (red solid), 0.75 sun (red dashed) and 0.6 sun (red dotted) illumination. All scans were collected at a scan rate of 100 mV s^{-1} with 0.5 M $LiClO_4$ in DMF as the supporting electrolyte.

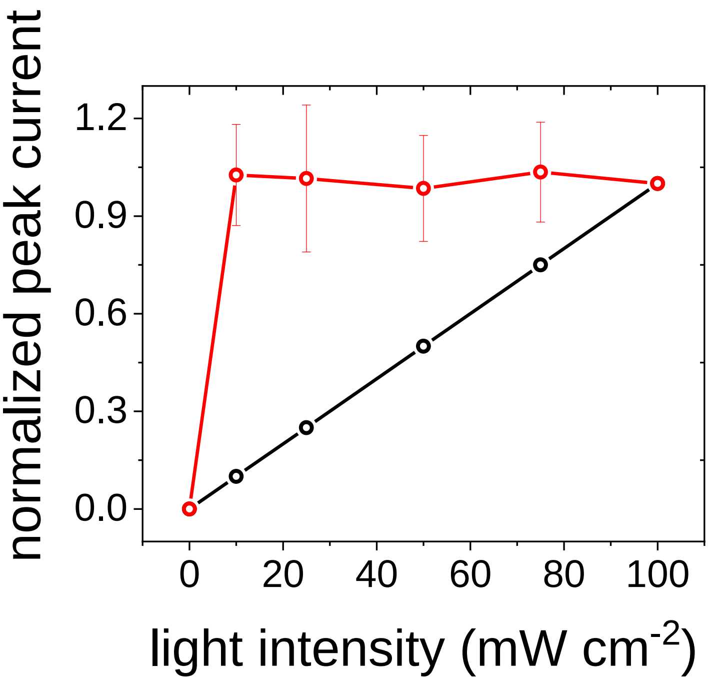

Supplementary Figure 19. Normalized theoretical (black) and actual (red) peak current densities for Zr(dcphOH-NDI)|TiO_2@Si working electrodes at varied illumination. Peak current is normalized to peak currents under 1-sun illumination to account for sample-to-sample film thickness variation. Theoretical peak current is calculated assuming the current is photon-limited.

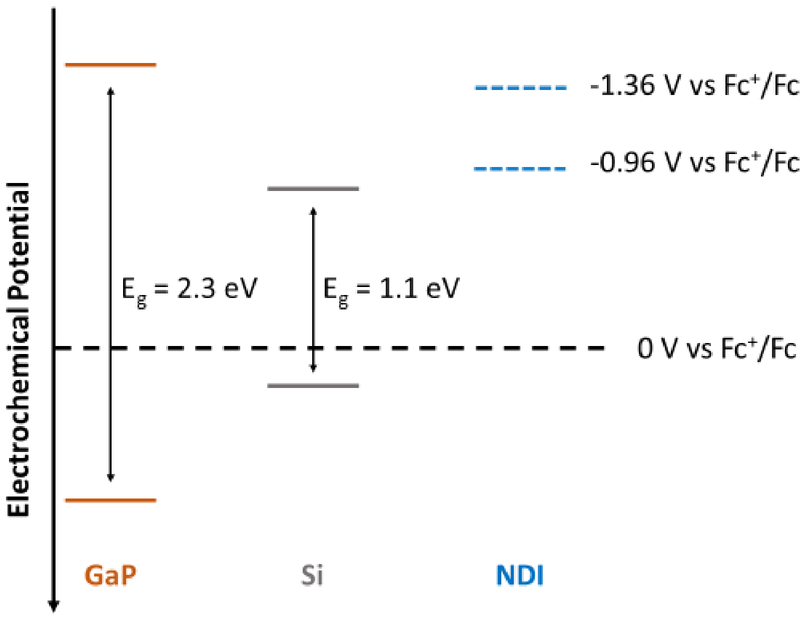

Supplementary Figure 20. Relative energy levels of photocathode components.

Supplementary Tables

Supplementary Table 1. Summary of XPS Zr:N ratios of Zr(NDI)@SC

	**Ratio Zr:N**
**Sample 1, spot 1 (Si)**	1:2.0
**Sample 1, spot 2 (Si)**	1:1.4
**Sample 2, spot 1 (Si)**	1:1.6
**Sample 2, spot 2 (Si)**	1:1.4
**Sample 3, spot 1 (GaP)**	1:1.7
**Sample 3, spot 2 (GaP)**	1:1.7
**Sample 4, spot 1 (GaP)**	1:1.7
**Sample 4, spot 2 (GaP)**	1:2.0
**Sample 5, spot 1 (GaP)**	1:2.2
**Sample 5, spot 2 (GaP)**	1:1.9

Supplementary Table 2. Summary of EDX Zr:N ratios of Zr(NDI)@Si

	**Analysis Mode**	**Ratio Zr:N**
**Sample 1, spot 1**	Map	1:1.5
**Sample 1, spot 2**	Map	1:1.7
**Sample 1, spot 3**	Map	1:1.9
**Sample 2, spot 1**	Point & id	1:2.6
**Sample 2, spot 2**	Point & id	1:2.4
**Sample 3, spot 1**	Map	1:2.4
**Sample 3, spot 2**	Line	1:1.6

Supplementary Table 3. Elemental Composition of Zr(NDI)|TiO_2@GaP Determined by SEM-EDX

**Element**	**Atomic %**
C	48.9
Ga	17.7
P	16.1
O	11.8
N	3.3
Zr	2.2
Total	100

Supplementary Methods

The diffusion coefficient was calculated using the Cottrell equation (Supplementary Equation 1):

$$D_{app} = \left(\frac{slope \times \sqrt{\pi}}{C^0_{NDI} \times S \times F}\right)^2 \qquad \text{Supplementary Eq. 1}$$

where $slope$ is the slope of the Cottrell plot Q vs $\sqrt{t}$, F is Faraday's constant (C mol^{-1}), C^0_{NDI} is the concentration of NDI linkers on the surface (mol cm^{-3}), D_{app} is the apparent diffusion coefficient (cm^2 s^{-1}), and S is the surface area of the electrode (cm^{-2}). For one value of D_{app}, (1.6 x 10^{-10} cm^2 s^{-1}), the following values were used: $slope$ = 4.66 x 10^{-4}, C^0_{NDI} = 2.43 x 10^{-3} mol cm^{-3}, and S = 0.28 cm^2. Five values were averaged together to calculate the reported D_{app} and standard deviation.

This value of D_{app} was used to calculate the amount of film probed (eq. 2):

$$\delta = \sqrt{\frac{D_{app}RT}{F\upsilon}}$$

using D_{app} = 10^{-10} cm^2 s^{-1} and υ = 0.005 V, the film diffusion layer is 226 nm.

The theoretical peak current was calculated using a finite diffusion model:

$$i_{pc} = FSC^0_{NDI}d_f\frac{F\upsilon}{4RT}$$

using S = 0.28 cm^2, C^0_{NDI} = 4.53 x 10^{-3}, d_f = 150 nm, and υ = 0.005 V. Dividing the result by the surface area of the electrode, S, gives a current density, j_{pc}, of 0.32 mA cm^{-2}. Five values were averaged together to calculate the reported i_{pc} and standard deviation.

Supplementary Reference

1. Johnson, B. A., Bhunia, A., Fei, H., Cohen, S. M. & Ott, S. Development of a UiO-Type Thin Film Electrocatalysis Platform with Redox-Active Linkers. *J. Am. Chem. Soc.* **140**, 2985–2994 (2018).
